# Nintedanib in Combination With Chemotherapy in the Treatment of Non-small Cell Lung Cancer: A Systematic Review and Meta-Analysis

**DOI:** 10.7759/cureus.53812

**Published:** 2024-02-07

**Authors:** Abdulhameed Alhadeethi, Sara Adel Awwad, Mohamed Abed, Ahmed Mostafa Amin, Menna M Aboelkhier, Mazen Negmeldin Aly Yassin, Maha H Morsi, Muataz Omar Kashbour

**Affiliations:** 1 Department of General Medicine, Medical Research Group of Egypt, Negida Academy LCC, Arlington, USA; 2 Department of General Medicine, Al-Salam Teaching Hospital, Mosul, IRQ; 3 College of Medicine, Jordan University of Science and Technology, Irbid, JOR; 4 Department of Internal Medicine, Faculty of Medicine, University of Tripoli, Tripoli, LBY; 5 Department of Internal Medicine, Faculty of Medicine, Al-Azhar University, Cairo, EGY; 6 Department of Internal Medicine, Faculty of Science, Cairo University, Cairo, EGY; 7 College of Medicine, Helwan University, Cairo, EGY; 8 Department of Oncology, Medical Research Group of Egypt, Negida Academy LLC, Arlington, USA; 9 Department of Chemical Pathology, Misr University for Science and Technology, Giza, EGY; 10 Department of Diagnostic Radiology, National Cancer Institute, Misrata, LBY; 11 Department of Radiology, Medical Research Group of Libya, Negida Research Academy, Arlington, USA

**Keywords:** non-small cell lung carcinoma (nsclc), vegfr inhibitor, meta-analysis, lung neoplasms, nintedanib

## Abstract

Lung cancer remains a major global health challenge, contributing to substantial morbidity and mortality rates. Nintedanib, a tyrosine kinase inhibitor, has demonstrated potential as a treatment for lung cancer. We aim to evaluate nintedanib's efficacy in treating patients with non-small cell lung cancer (NSCLC), depending on the available evidence. Our search for relevant articles was conducted on PubMed, Cochrane Library, Scopus, and Web of Science for randomized controlled trials (RCTs) that involved adult patients with NSCLC up to August 15, 2023. These trials compared the combination of nintedanib and chemotherapy to either placebo plus chemotherapy or chemotherapy alone. Our main outcomes include progression-free survival (PFS) and overall survival (OS). We utilized the Review Manager Software V.5.4 (The Cochrane Collaboration) to analyze all relevant data. Three identified trials, which included 2270 patients, fulfilled the inclusion criteria. Our analysis showed significantly improved PFS (hazard ratio (HR) = 0.79; 95% confidence interval (CI) 0.71-0.88, P < 0.0001) in patients receiving nintedanib compared to placebo. However, OS was not statistically significant (HR = 0.96; 95% CI 0.88-1.05, P = 0.35). In conclusion, a combination of nintedanib and chemotherapy in treating patients with NSCLC was associated with improved PFS than chemotherapy alone but not with improved OS. Further clinical trials assessing nintedanib in the setting of NSCLC are necessary before any further recommendations can be made.

## Introduction and background

Lung cancer has one of the poorest five-year survival rates among all cancer types and stands as the leading cause of cancer-related fatalities across the globe [1,2]. It comprises around 12% of all newly identified cancer cases annually [3]. Lung cancer can be categorized into three primary types: non-small cell lung cancer (NSCLC), small-cell lung cancer (SCLC), and lung carcinoid tumors. About 85% of all new cases of lung cancer are diagnosed with NSCLC, which presents a significant healthcare challenge worldwide due to its tendency to manifest clinical symptoms and receive diagnosis at later stages [4,5]. In fact, 57% of NSCLC patients are detected with advanced forms (Sage IV) of this illness [5].

Angiogenesis and the formation of new blood vessels play a role in the tumor pathogenesis, progression, and metastasis. In the context of NSCLC, heightened angiogenesis has been linked to more advanced stages of the disease and less favorable clinical outcomes [6,7]. Platinum-based chemotherapy has traditionally been the standard treatment for individuals with advanced or metastatic NSCLC who do not have identifiable targetable genetic mutations [8]. While most NSCLC patients initially experience disease stabilization with first-line platinum-based therapy, nearly all face disease progression within a year after completing this initial treatment and necessitate second-line therapy [5,9]. Currently approved options for second-line NSCLC treatment include single-drug therapies like docetaxel, gemcitabine, pemetrexed, or erlotinib [5,9,10]. Numerous studies have explored the safety of nintedanib, an oral triple angiokinase inhibitor that can block RET, FLT3, and members of the SRC family, as a treatment choice for NSCLC patients [11-13].

In a phase III double-blind, randomized controlled trial, Reck et al. [11] found that advanced NSCLC patients who received nintedanib combination chemotherapy had longer overall survival (OS) and progression-free survival (PFS). Additionally, findings from a network meta-analysis conducted by Popat et al. have corroborated these results, highlighting the efficacy of nintedanib combination therapy alongside chemotherapy compared to chemotherapy alone for patients with advanced NSCLC [14]. In a different study, a phase III double-blind, randomized controlled trial has reported limited benefits in OS among patients afflicted with refractory, advanced NSCLC [12].

In light of the importance of this subject matter and the conflicting findings present in the available data, we conducted this meta-analysis. Our objective was to fill the existing knowledge void and offer valuable insights regarding the safety and effectiveness of combining nintedanib with chemotherapy for the treatment of NSCLC patients.

## Review

Methods

For this meta-analysis, we followed the Preferred Reporting Items for Systematic Reviews and Meta-Analysis (PRISMA) guidelines [15] and registered the study in PROSPERO (CRD42023451851) to ensure transparency and methodological rigor.

Literature Search

As part of our extensive literature search, we searched several databases, including Scopus, PubMed, the Cochrane Library, and Web of Science. The most recent search was conducted on August 15, 2023. We used a combination of Medical Subject Headings [MeSH] phrases to build our search strategy, including "Nintedanib OR BIBF 1120 OR VEGFR inhibitor," and "chemotherapy OR antineoplastic agents," and "Lung neoplasms OR non-small cell lung cancer OR small cell lung cancer." As part of our rigorous approach, we manually searched the reference lists of studies that fulfilled our initial criteria to ensure that no potentially suitable papers were missed during our search process. Further details can be found in Table 1.

**Table 1:** Details of the search strategy of the databases. WOS: Web of Science

Database	Keywords	NO.
PubMed	(nintedanib OR BIBF 1120 OR VEGFR inhibitor) AND (chemotherapy OR antineoplastic) AND (lung neoplasms OR non-small cell lung cancer OR small cell lung cancer)	1112
Scopus	(nintedanib OR (BIBF AND 1120) OR (VEGFR AND inhibitor)) AND (chemotherapy OR antineoplastic) AND ((lung AND neoplasms) OR (non-small AND cell AND lung AND cancer) OR (small AND cell AND lung AND cancer))	1232
WOS	(nintedanib OR BIBF 1120 OR VEGFR inhibitor) AND (chemotherapy OR antineoplastic) AND (lung neoplasms OR non-small cell lung cancer OR small cell lung cancer)	236
Cochrane	(nintedanib OR BIBF 1120 OR VEGFR inhibitor) AND (chemotherapy OR antineoplastic) AND (lung neoplasms OR non-small cell lung cancer OR small cell lung cancer)	111

Rayyan software (Qatar Computing Research Institute, Doha, Qatar) [16] was used during the selection process; after removing the duplication, the studies were screened by two different reviewers using only the titles and abstracts. After this first screening, the full texts of the studies that were thought to be possibly eligible underwent a rigorous evaluation process before being finally included in the review. When the two review team members could not agree on whether a specific study qualified or not, they had a discussion to come to an agreement. A third reviewer made the final decision if an agreement was not reached.

Selection and Eligibility Criteria

In the process of selecting articles for inclusion in our analysis, we adhered to a specific set of criteria outlined by PICOS. Participants: We considered studies that involved patients with NSCLC of any staging and pathological type. This inclusivity allowed us to encompass a broad spectrum of NSCLC patients in our review. Interventions: Our focus was on studies where nintedanib was administered with chemotherapy. This particular combination was of primary interest to evaluate its efficacy. Comparator: We considered studies with a comparator arm using either chemotherapy alone or chemotherapy with a placebo, which allowed us to gauge the relative effectiveness of nintedanib in the treatment of NSCLC. Outcomes: Our analysis included a number of key outcomes. The primary outcomes of interest were PFS and OS. In addition, we considered secondary outcomes, which included the objective response rate (ORR) and disease control rate (DCR). OS measures the OS time from the time of treatment initiation to the time of death from any cause, while PFS measures the time from the time of treatment initiation to the time of disease progression or death. ORR is implicated in tumor response to drugs, while DCR is the percentage of patients whose disease shrinks or remains stable over a certain time period. Design: We focused exclusively on RCTs that met specific criteria. These RCTs needed to be available as full-text articles and published in peer-reviewed journals.

Our selection process excluded several types of studies, including nonrandomized studies, single-arm studies, observational studies, animal experiments, studies that did not report relevant outcome data, and secondary analysis studies or only abstracts without full text.

Data Extraction

The following data were extracted independently by two investigators using a standardized Google sheet: study characteristics (study ID, year of publication, country, number of participants, cancer type, and type of intervention), participant characteristics (age, sex, smoking state, stage of SCLC, presence of brain metastasis, and Eastern Cooperative Oncology Group status), and outcome measures (PFS, OS, ORR, and DCR). Another investigator resolved any differences and revised the data for more precision.

Risk of Bias Assessment

To evaluate the quality and potential bias in the RCTs under consideration, we applied the Cochrane risk of bias tool 2, denoted as ROB2 [17]. This tool facilitated a comprehensive evaluation of several critical domains in each trial. To ensure a rigorous and unbiased assessment, two independent reviewers examined the seven essential items of this tool. In our commitment to thoroughness and objectivity, a third reviewer was consulted when necessary to provide supplementary input and resolve any discrepancies or disagreements. We did not perform a visual assessment of comparison-adjusted funnel plots to detect publication bias as the number of studies included was insufficient [18].

Statistical Analysis

For the comprehensive analysis of the extracted data, we employed RevMan version 5.5, a software tool developed by the Cochrane Collaboration. We determined the risk ratio (RR) and their corresponding confidence interval (CI) using the Mantel-Haenszel method for dichotomous outcomes. In addition, utilizing either fixed or random models in our analysis was contingent on the outcomes of two critical statistical tests: the Q-test and the I-squared test. If the I-squared test indicated significant heterogeneity, with an I-squared value surpassing 50%, and the p-value (P) from the Q-test was less than 0.1, we selected random models. These models are particularly helpful when dealing with significant differences between studies. Furthermore, we established the criterion for determining statistical significance when p-values were less than 0.05.

Results

Literature Search and Study Selection

**Figure 1:**
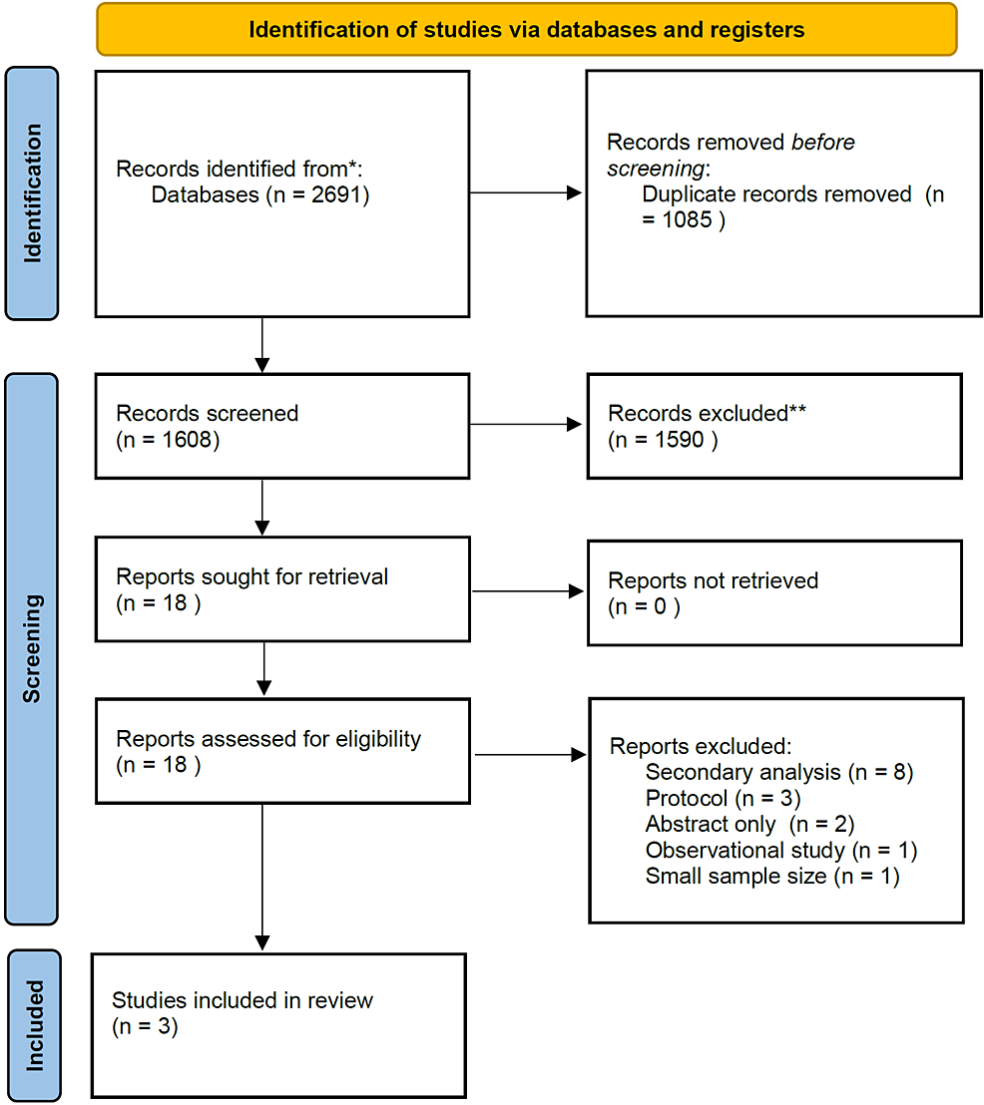
PRISMA flow diagram of selection of studies. PRISMA: Preferred Reporting Items for Systematic Reviews and Meta-Analyses

This exhaustive search yielded 2691 entries. We reviewed the titles and abstracts of the remaining 1608 records, applying stringent eligibility criteria to identify studies that met our research objectives. This rigorous screening process led us to a narrower selection of 18 studies that were relevant to our investigation. Subsequently, these 18 studies underwent full-text screening to ascertain their suitability for inclusion in our meta-analysis. Ultimately, after further evaluation, only three of these studies were suitable and were thus incorporated into our meta-analysis. The selection procedure is shown in Figure 1.

Characteristics of Included Studies

Three studies were included in our meta-analysis; all were multicenter and double-blinded, except for Otsubo et al., which was an open-label study. The studies were published between 2014 and 2022 and had a total of 2270 patients. The patients were randomized to receive nintedanib plus chemotherapy as an intervention or placebo plus chemotherapy as a control, apart from Otsubo et al., where the comparator was chemotherapy alone without a placebo. The chemotherapy of choice in Reck et al. was docetaxel, while pemetrexed was used in Hanna et al., and carboplatin plus nab-paclitaxel in Otsubo et al. Table 2 shows a summary of the included studies, and Table 3 shows the baseline demographic characteristics of included studies.

**Table 2:** Summary of included studies. NSCLC: Non-small cell lung cancer; PFS: progression-free survival; OS: overall survival; ORR: objective response rate; DCR: disease control rate; IPF: idiopathic pulmonary fibrosis

Study ID	Location	Year	Sample size	Cancer type and stage	Treatment groups: Intervention Comparator		Median follow-up (Months)	Main results	Author conclusion
Reck et al., 2014 [11]	211 centers in 27 countries	2014	1314	Stage IIIB/IV recurrent NSCLC	Docetaxel plus nintedanib	Docetaxel plus placebo	31·7	PFS, OS, ORR, and DCR	The combination of nintedanib and docetaxel proves to be an effective second-line treatment for advanced NSCLC
Hanna et al., 2016 [12]	202 centers in 32 countries	2016	713	Stage IIIB/IV recurrent, non-squamous NSCLC	Nintedanib–pemetrexed	Placebo– pemetrexed	26.8	PFS, OS, ORR, and DCR	The combination of nintedanib with pemetrexed demonstrated a notable extension in PFS for patients with advanced NSCLC
Otsubo et al., 2022 [19]	Japan	2022	243	Stage IIIB/IV recurrent NSCLC	Nintedanib plus chemotherapy (carboplatin plus nanoparticle albumin-bound paclitaxel [nab-paclitaxel])	Chemotherapy alone	12.1	PFS, OS,ORR	Nintedanib in combination with chemotherapy improved overall survival in patients with non-squamous histology.

**Table 3:** Baseline demographic characteristics of included studies. ECOG: Eastern Cooperative Oncology Group

Author, year	Treatment groups	No. of participants	Age (years)	Male (%)	Smoking history (%)		ECOG performance status (%)		Brain metastases at baseline (%)	
					Current/ex- smoker	Never	0	1	Yes	No
Reck et al., 2014 [11]	Intervention	655	60 ± 10.4	72.7	74.8	25.2	28.5	71.3	5.8	94.2
	Control	659	60 ± 8.92	72.7	75.6	24.4	28.7	71.3	5.8	92.87
Hanna et al., 2016 [12]	Intervention	353	60 ± 10.5	55.2	69.12	30.9	38.2	61.8	10.2	89.8
	Control	360	59 ± 10	57.8	66.11	33.9	38.6	61.4	10	90
Otsubo et al., 2022 [19]	Intervention	121	71 ± 4.83	89.3	95	5	33.9	66.1	NR	NR
	Control	122	71 ± 4.17	91	97.5	2.5	46.7	53.3	NR	NR

Quality Assessment

**Figure 2:**
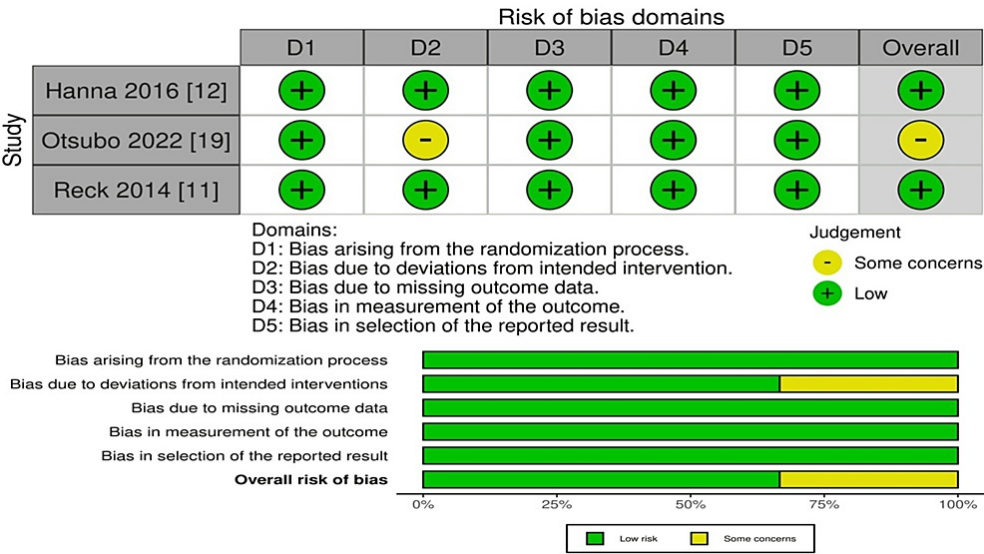
Risk-of-bias assessment for the included studies.

The assessment of the studies that we included in our analysis for their quality is illustrated in Figure 2. Using the Cochrane risk-of-bias 2 (ROB2) tool for randomized trials, our assessment revealed that two of the studies had a low risk of bias, while one study was identified with a moderate risk.

Primary Outcomes

**Figure 3:**
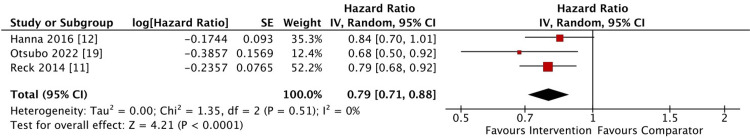
Forest plot comparing the progression-free survival.

Efficacy evaluation of PFS: PFS improved significantly in the nintedanib plus chemotherapy group when compared to the comparator group. This difference held significant statistical value (HR = 0.79; 95% CI [0.71 to 0.88]; P < 0.0001). Furthermore, when considering the combined data from these studies, the results were consistent and showed homogeneity, as indicated by the random-effects model (I² = 0%; P = 0.51) (Figure 3).

**Figure 4:**
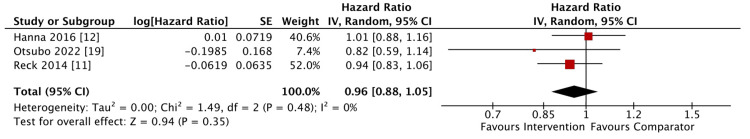
Forest plot comparing the overall survival.

Efficacy evaluation of OS: The effect of OS between the two groups was examined by pooling the data from all three trials. The OS was not significant between both groups (HR = 0.96; 95% CI [0.88 to 1.05]; P = 0.35), and the studies were pooled according to the random-effects model (I² = 0%; P = 0.48) (Figure 4).

Secondary Outcomes

**Figure 5:**
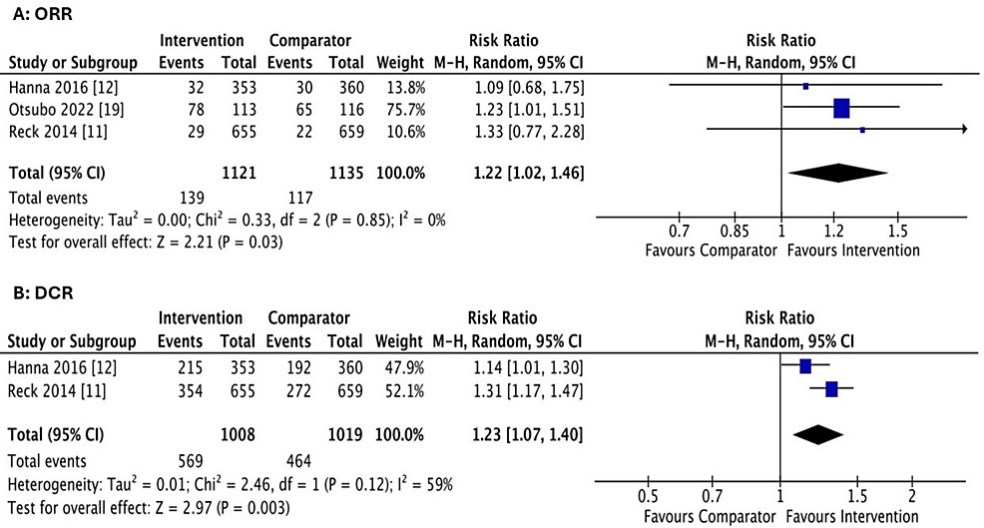
A: Forest plot comparing the objective response rate (ORR). B: Forest plot comparing the disease control rate (DCR) .

Efficacy assessment of ORR & DCR: All the studies reported ORR. The nintedanib plus chemotherapy group showed higher ORR than the comparator group, with the results being statistically significant (RR = 1.22; 95% CI [1.02 to 1.46]; P = 0.03; I^2^= 0%). For DCR, only Hanna et al. and Reck et al. reported DCR favoring the nintedanib plus chemotherapy group over the comparator group. The results were statistically significant (RR = 1.23; 95% CI [1.07 to 1.40]; P = < 0.003, I^2 ^= 52%) (Figure 5).

Discussion

We conducted this meta-analysis of 2270 patients to gain insight into the efficacy of nintedanib in combination with chemotherapy. Based on preclinical research findings, nintedanib has demonstrated its efficacy as a potent triple angiokinase inhibitor. As a result, it exerts its influence by targeting three critical angiogenic pathways that play crucial roles in promoting the growth of blood vessels. Specifically, nintedanib acts on the pathways that are regulated by vascular endothelial growth factor (VEGF), fibroblast growth factor receptors (FGFRs), and platelet-derived growth factor receptors (PDGFRs), and these data revealed significant suppression of tumor development by nintedanib in human NSCLC xenograft models, as well as other tumors [20]. As a result, quite a few clinical studies have been conducted using this combination therapy. There remains uncertainty regarding the value of combination therapy compared to chemotherapy alone. However, in NSCLC, a number of clinical trials are assessing the safety and effectiveness of immunotherapy in conjunction with antiangiogenic drugs. Based on the potential synergistic antitumor effects and preliminary findings, this combination treatment appears to be effective and well-tolerated [21,22].

In our current analysis, we observed a substantial enhancement in PFS among patients who underwent nintedanib treatment in comparison to those who received placebo plus chemotherapy or chemotherapy alone. These results were subsequently validated in a network meta-analysis, focusing on including nintedanib alongside docetaxel for individuals with refractory NSCLC [14]. Their research determined that introducing nintedanib alongside docetaxel potentially outperforms using docetaxel alone concerning PFS and OS. However, our present meta-analysis did not reveal any distinction in OS between the two treatment cohorts. PFS events occur earlier in the course of the disease than OS events. Detecting significant differences in OS typically requires a sufficient number of events (such as deaths) to occur within the studied population. If the studies have a relatively short follow-up duration, as a study included in our analysis conducted by Otsubo et al. [19] (median follow-up time was 12.1 months), it may be difficult to detect statistically significant differences in OS.

However, there has been no significant survival benefit associated with previous antiangiogenic agents that have been evaluated in the second-line setting despite PFS improvements [23,24]. Additionally, a number of other trials have examined antiangiogenic compounds in the first-line setting in conjunction with chemotherapy; also, they have not succeeded in showing any influence on OS in NSCLC patients [25,26].

In the present study, ORR ​​is barely significant [RR = 1.22; 95% CI [1.02 to 1.46]; P = 0.03]. However, our findings align with the ZODIAC study [27] and ZEAL study [28]. In the ZODIAC trial, the combination of docetaxel and vandetanib resulted in a notable enhancement in the ORR among patients with advanced NSCLC compared to those who received chemotherapy alone. Likewise, the findings from the ZEAL study corroborate our observations, demonstrating an increased ORR when pemetrexed is combined with vandetanib in NSCLC patients.

This study has some notable limitations that should be addressed in the discussion. The studies began by administering varying treatment doses to patients, leading to significant variability in the results. Moreover, the patient population included individuals with different types of lung cancer, each characterized by distinct disease stages and varying levels of severity. This heterogeneity within the patient cohort can complicate the interpretation of treatment outcomes, as the response to therapy may differ significantly between cancer subtypes and disease stages.

Furthermore, one of the included trials focused on patients with both lung cancer and idiopathic pulmonary fibrosis. This dual diagnosis presents a distinct clinical scenario, and the findings from this subgroup may not be directly generalizable to the broader lung cancer population. Future clinical trials should consider longer follow-up durations to better assess the impact of nintedanib on PFS and OS. This would provide a more comprehensive understanding of its true clinical benefit. It should also explore nintedanib in combination with other agents or modalities, including immunotherapy, to identify patient subgroups for personalized treatment strategies. Our findings mark a step forward in the quest to optimize the NSCLC treatment.

## Conclusions

The study findings suggest that the combined use of nintedanib and chemotherapy in NSCLC patients leads to enhanced PFS compared to chemotherapy alone. However, despite this improvement, no significant improvement in OS was observed. To provide more nuanced insights and recommendations, it is imperative to conduct additional clinical trials specifically examining nintedanib within the context of NSCLC. This approach will contribute to a more comprehensive understanding of the treatment's efficacy and inform future clinical strategies.
